# Comparison of the Effects of Adenosine, Inosine, and Their Combination as an Adjunct to Reperfusion in the Treatment of Acute Myocardial Infarction

**DOI:** 10.5402/2012/326809

**Published:** 2012-03-14

**Authors:** Abdel Shafy, Vincent Molinié, Miguel Cortes-Morichetti, Vincent Hupertan, Nermine Lila, Juan C. Chachques

**Affiliations:** ^1^Laboratory of Biosurgical Research, Alain Carpentier Foundation, University Paris Descartes, 75015 Paris, France; ^2^Department of Pathology, Saint Joseph Hospital, 75014 Paris, France

## Abstract

Adenosine and inosine are both key intracellular energy substrates for nucleotide synthesis by salvage pathways, especially during ischemic stress conditions. Additionally they both possess cell protective and cell repair properties. The objective of this study is to detect potential advantages of the combination of adenosine and inosine versus each drug alone, in terms of ventricular function, infarct size reduction and angiogenesis. Myocardial ischemia was created in rodents and treated with adenosine, inosine or their combination. Results of experiments showed that the combination of both drugs significantly reduced infarct size and improved myocardial angiogenesis and ventricular function. The two compounds, while chemically similar, use different intracellular pathways, allowing for complementary biological activities without overlapping. The drug combination at specific 1 : 5 adenosine : inosine dose ratio demonstrated positive cardiologic effects, deserving further evaluation as an adjunct to reperfusion techniques during and after acute coronary syndrome. The association of adenosine and inosine may contribute to reduce myocardial infarction morbidity and mortality rates.

## 1. Introduction

*Adenosine* has demonstrated cell-protective activity in animal and human models of ischemia reperfusion that involves the participation of all adenosine receptor subtypes [1, 2]. Adenosine also stimulates the production of vascular endothelial growth factor (VEGF) which promotes angiogenesis, especially during and immediately after acute ischemia [3–5].

*Inosine* is an inotropic agent without chronotropic effect that does not increase myocardial oxygen consumption [6–9]. It has also been demonstrated in experimental models cell-protective activities [10, 11]. Whereas the effects of adenosine are mediated by the combined function of the entire adenosine receptor family (A1, A2a, A2b, A3), no specific inosine receptor has been identified to date at the level of vascular and cardiac cells. Therefore, pathways supporting its effects remain uncertain and most of them are presumed to be energy restoration mechanisms [11].

A combination (mixture) of the two nucleosides adenosine and inosine at a specific 1 : 5 dose ratio has been found to synergistically enhance arterial vasodilation and be haemodynamically equivalent to adenosine optimal dose alone, using a dose of adenosine cut by 50%.

Given, firstly, that ATP and energy nucleoside substrates depletion is a rapid process and the major cause of death in myocardial cells submitted to acute ischemic conditions, and, secondly, that adenosine and inosine both possess cell protective and repair activities, we found interesting to assess in an acute coronary syndrome model with reperfusion injury, the potential therapeutic value of their combination at optimal hemodynamic adenosine : inosine dose: ratio of 1 : 5.

The objective of the present study is to detect the existence, in a widely used acute ischemia experimental model, of potential advantages of the combination of adenosine and inosine versus each drug alone, in terms of ventricular function, infarct size reduction, and angiogenesis.

## 2. Materials and Methods

After creation of a left ventricular (LV) acute myocardial infarction in rats followed by reperfusion, adenosine and inosine drugs used alone or in combination were intravenously infused for one hour. Evaluation included LV function assessment by echocardiography and histological studies of myocardial tissue.

### 2.1. Products

Both adenosine (Adenosine A9251) and inosine (Inosine I4125) were obtained from Sigma-Aldrich Chemical, Evry, France.

### 2.2. Doses Finding and Search for an Optimal Adenosine : Inosine Dose Ratio

Search for a correlation between doses in the rat and those employed in the human was a prerequisite for this experiment.

In a first series of 5 animals (all excluded from the ACS protocol), carotid blood flow measurements were used to determine which dose in the rat induces maximal carotid blood flow and correlates to the dose described in the man, that is, 120–140 *μ*g/kg/mn [12, 13]; from there the dosage that in the rat approximately corresponds to adenosine 50–70 *μ*g/kg/mn, as had been IV given to patients with ACS in several human trials [14–16], was easy to estimate. We used starting doses drawn from the data published by Ohnishi et al. [17] about the effect of adenosine on blood pressure measured at the carotid artery level in the normotensive rat, because no reference specifically focused on CaBF after adenosine administration could be found in the literature with this animal. Finally the fixed dose of adenosine 0.05 mg/kg/mn was identified in the rat as equivalent to 50–70 *μ*g/kg/mn in the human.

In further series of rats (5 per group, six groups) incremental doses of inosine were combined with this fixed dose of adenosine (equivalent to 50% adenosine max) and administered intravenously; the effect of these combinations on CaBF was then compared to that of adenosine max. This methodology allowed us to select 1 : 5 as the optimal adenosine : inosine dose: ratio combination providing maximal CaBF.

### 2.3. Myocardial Ischemia/Reperfusion Injury

In Wistar rats (weight 300 ± 30 g) a left ventricular myocardial ischemia was surgically created by transitory left coronary artery surgical occlusion (45 minutes), followed by reperfusion. All procedures were approved by the French National Institute of Health and Medical Research and received care in compliance with the European Conventions.

### 2.4. Anesthesia

Rats were anesthetized with a mixture of ketamine (Panpharma, Fougeres, France) 50 mg/kg and acepromazine (Vetranquil, Sanofi-Aventis, Libourne, France) 2 mg/kg, using intramuscular injections. An endotracheal metallic tube was inserted through the mouth for artificial respiration using air, maintained with a Harvard Rodent Ventilator model 683 (frequency 85 cycles/min, tidal volume 20 mL).

### 2.5. Surgical Procedures

In the supine position, a left antero-lateral thoracotomy was performed via the fourth intercostal space. The pericardium was opened and the left coronary artery and its branches were localized using magnifying glasses. A 6-0 nonabsorbable Prolene suture was passed underneath the left coronary artery (just proximal to their division), the flow was interrupted using a 5F polyurethane occluder. This occluder was released after 45 minutes, therefore the myocardial ischemic territory was reperfused. Significant EKG changes, including widening of the QRS complex and elevation of the ST segment, and color changes of the area at risk were considered indicative of coronary occlusion. The intercostal space was then closed with a 6-0 nonabsorbable Prolene suture and the skin with a 5-0 absorbable Vicryl suture. One hour after closure baseline echocardiography was performed. The animals with a baseline LV ejection fraction higher than 30% were eliminated from the study. Afterwards the femoral vein was surgically exposed, a 24 G, 18 mm PTFE catheter (Intrafon 2 Vygon, Ecouen, France) was inserted for infusion of a therapeutic solution during 60 minutes using an electrical infusion pump.

### 2.6. Treatment Groups

After creation of myocardial ischemia, animals were randomized for IV infusion of:

*Group 1 *(*n* = 12): saline (sham group);

*Group 2 *(*n* = 12): adenosine at the dose of 0.05 mg/kg/min;

*Group 3 *(*n* = 12): inosine at the dose of 0.25 mg/kg/min;

*Group 4 *(*n* = 12): combination of adenosine at the dose of 0.05 mg/kg/mn with inosine at the dose of 0.25 mg/kg/min (1 : 5).

### 2.7. Functional Evaluation

Echocardiography studies were performed using a Sequoia Acuson system 512 (with a 15 MHz probe), one hour after myocardial ischemia (baseline) and at postoperative day 45. Echocardiography assessed LV ejection fraction (EF), end-systolic, and end-diastolic ventricular diameters and volumes.

### 2.8. Histological Studies

After echocardiography rats were killed at postoperative day 45. The hearts were fixed in 4% formaldehyde solution for 48 h, and then dissected. For each case numerized photographic was obtained before and after dissection. After fixation the hearts were cut transversally in the middle third between base and apex in sections of 2 mm and paraffin embedded. For histological analysis 5 *μ* tissue section performed and a usual HES staining was performed.

#### 2.8.1. Immunohistochemistry

It was performed using marketed monoclonal antibody antifactor VIII (1/400, Dako). Briefly, tissue sections were antigen-retrieved in 0.1 M citrate buffer, pH 6, in a hot steamer for 30 min. The immunostaining process was performed on a Dako Stainer processor at ambient temperature. Subsequently, slides were incubated with the antifactor VIII antibody, biotinylated secondary anti-mouse/anti-rabbit mixed antibody with the same avidine-peroxidase complex (Dako), and chromogenic substrate diaminobenzidine; slides were then counterstained with hematoxylin. Capillary density was evaluated on HES slides after Factor VIII immunohistostaining.

### 2.9. Infarct Size Evaluation

For each case a percentage infarction of the LV surface was evaluated by a millimetric scale section (square surface of infarction). For each section the surface of infarction was calculated using a micrometer (Lmax × lmax). The infarct volume was evaluated by the number of infarcted section observed.

### 2.10. Angiogenesis Index

An angiogenesis index was defined and calculated. A semiquantitative method was used to score angiogenesis in the infarct area. To reduce inter-animal variations we arbitrarily deemed more relevant to use a scale considering a 5 capillaries difference/animal rather than the total number of capillaries per group:

no arterial capillary = 0;
less than 5 capillaries = 1;
between 5 and 10 = 2;
between 10 and 15 = 3;
over 15 = 4.

The angiogenesis index was calculated as the mean of the total angiogenesis score per group. As for an example the total angiogenesis score for the inosine-alone group was calculated as the sum of all angiogenesis scores (one per rat) divided by the number of animals (*n* = 12): 1 + 3 + 2 + 4 + 2 + 2 + 3 + 3 + 3 + 2 + 3 + 4 = 32/12 = 2.6.

### 2.11. Statistical Analysis

An exploratory analysis was first conducted to study the distribution of the variables using the Kolmogorov-Smirnov test and graphical methods. Homoscedasticity was also studied via the Levene test. In case of heteroscedasticity the Welch's ANOVA was used rather than the one-way ANOVA analysis of variance (in this situation ANOVA may yield inaccurate *P* values). When variables showed abnormal distribution the *H* test of Kruskal Wallis (nonparametric equivalent of ANOVA) was employed.

To further conduct pairwise comparisons (in case of significant *P* values with Welch's Anova or Kruskal Wallis test), we used the C Dunnet test (if heteroscedascity) or the nonparametric version of the Student-Newman-Keuls (SNK) for multiple comparisons. The C Dunnet test and SNK were preferred to other methods to avoid overestimate of the *P*-value when performing multiple comparison studies. A 0.05 level was considered significant for all the tests. Statistical procedures were performed using IBM SPSS Statistics 18.

## 3. Results

### 3.1. Mortality Rate

The operative mortality rate of our ischemia-reperfusion model was 11%. The mortality rate during the follow-up period was 3%. Animals included into the study to replace dead animals were randomly assigned to one group or the other until the final number of survivors reaches 12 in each group.

After the double-blind code was broken the mortality rate during the whole study (perioperative + follow-up periods) appeared slightly higher in the sham (7%) and adenosine (8%) groups as compared to the inosine (3%) and drug combination (3%) groups.

### 3.2. Functional Evaluation

Echocardiography studies showed that LVEF was similarly preserved 45 days after myocardial infarction in the treated rat groups by either adenosine, inosine or their combination, as compared to the sham group. However, a significant difference was demonstrated in the drug combination and adenosine alone groups versus sham, but not between inosine and the sham group (Table 1).

### 3.3. Histological Diagnosis

In the adenosine group the mean infarct size was 2.60 mm^3^ and the angiogenesis median index was 2. In the inosine group the mean infarct size was 2.62 mm^3^ and the angiogenesis median index was 3. In the adenosine/inosine combination group the mean infarct size was 1.70 mm^3^ and the angiogenesis median index was 4. For the control group the mean infarct size was 3.79 mm^3^ and the angiogenesis median index was 1.5. A significant difference was observed regarding infarct size between the drug combination and adenosine groups and the sham group (Table 2).

**Figure 1:**
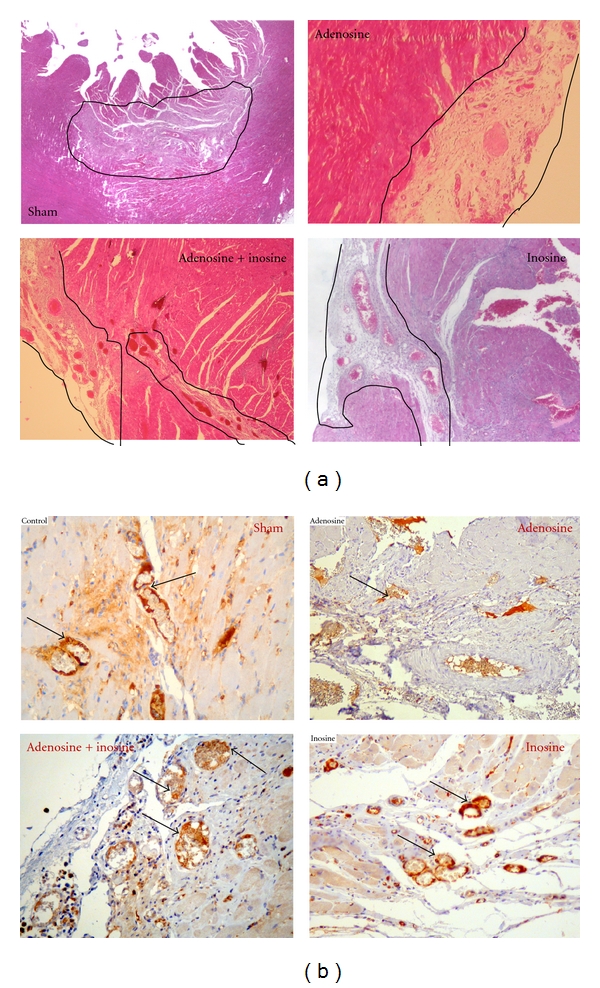
Histological analysis: focus of significant infarct scar (surrounded by black lines). Comparative angiogenesis in the infarct area, capillary density is on average higher in the combination group compared to other groups, 1: sham, 2: combination, 3: inosine, 4: adenosine. (a) Staining technique HES (magnification: Obj. ×20). (b) Immunohistochemistry using antifactor VIII (magnification: Obj. ×40). Arrows show capillaries neovascularisation.

On histologic analysis we found that inosine, adenosine, and drug combination groups had a proangiogenic effect superior to the sham and in particular that adenosine/inosine combination, besides being significantly better than the control, was also significantly superior to adenosine in this regard. (Figures 1(a) and 1(b)).

## 4. Discussion

*Adenosine* is a strong coronary vasodilator, and for this reason is used worldwide as a pharmacological stressor for the imaging of myocardial perfusion and the detection of coronary artery disease [18, 19]. Adenosine's ability to suppress the biological pacemaker activity and to slow atrio-ventricular conduction is also used in clinical practice, for the treatment of supraventricular tachycardia [20]. In addition, adenosine also possesses well-documented cell-protective and cell-repair activities [1–5]. However, its protective and restorative effects have been more convincingly demonstrated in animal studies than in human clinical studies. Furthermore, adenosine has a poor tolerability; this remains a serious limiting factor that, until now, greatly contributed to restrict the scope of its medical applications, especially when prolonged infusion times are indicated (i.e., 0.5 to 3 hours) in conscious patients. Therefore, although referenced as a major cell-protective agent, adenosine has not yet been cleared for such purpose by the regulatory authorities and therefore remains under investigation.

*Inosine*, the primary byproduct of adenosine, has been long considered a relatively either inactive or weak metabolite (e.g., it is a weak coronary vasodilator, ten times less potent than adenosine) and has been somewhat “brushed aside,” not to say forgotten by the scientific cardiologist community during the last decades. Yet, it should be remember that inosine has been used as an inotropic agent for more than 20 years in several countries (France, Germany, Japan, Russia) due to its capacity for increasing cardiac output without chronotropic effect and increase of myocardial oxygen consumption [21–24]. However, with the appearance of new and more competitive inotropic drugs, it was no longer commercially sold after the early 1980s.

Interestingly, and as opposed to adenosine, inosine is extremely well tolerated in humans, so much so that in the countries that marketed it, it was the preferred drug for patients over 65 years old with heart failure [2]. Recent evidence indicates that extracellular inosine has potent abilities to protect and repair cells, due to anti-inflammatory properties, as well as the ability to stimulate various growth factors and restore cell energy [11, 25–28].

In addition, due to a competitive mechanism between the two molecules at the level of specific cell membrane transporters (“equilibrative nucleoside transporters 1 and 2”) [29], the concurrent administration of adenosine and inosine makes it possible for inosine to interfere with adenosine in real time, to modulate its plasma clearance in a dose-dependent fashion without losing adenosine immediate reactivity (side effects cease rapidly after cessation of the infusion) and thus to reduce adenosine doses without loss of efficacy and improved safety. The final effect may be achieved for long infusion times (0.5 to several hours) and cannot be performed as safely and accurately (in a steady-immediate fashion, second after second) by other existing molecules, thanks to adenosine and inosine short half-lives.

In the present study which was strictly focused on cell protection and angiogenesis end-points, we observed that the adenosine/inosine combination effects surpassed those of adenosine and inosine alone.

### 4.1. Adenosine and Inosine Pathways

**Figure 2:**
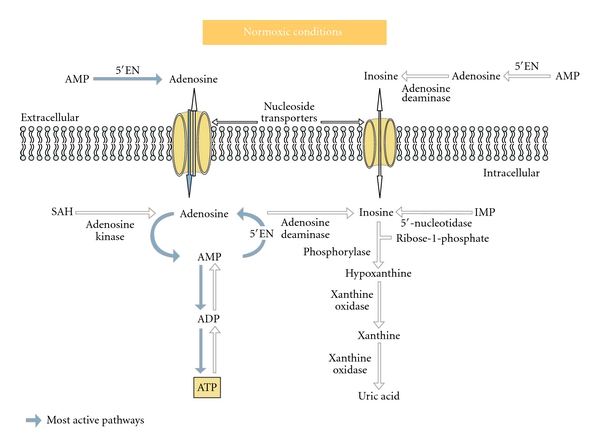
Adenosine and inosine pathways in normal conditions. During *“normoxia”* adenosine is released in small amounts at a constant basal rate partly from hydrolysis of S-adenosyl homocystein (SAH) by SA-homocysteinase and mostly by dephosphorylation of AMP by 5′ecto-nucleotidases (5′EN) localized in the cytosol or membrane-bound outside the cells. From there, intracellular adenosine is rephosphorylated to AMP by adenosine kinase and a small amount is released from the cell. Indeed the values of the Km of adenosine kinase for adenosine are between one and two orders of magnitude lower than those for the deaminase which is much less active. Therefore, it is thought that 5′-nucleotidase and adenosine kinase are simultaneously active so that a substrate cycle between AMP and adenosine is produced. Inosine production depends on adenosine basal production. It is formed by the deamination of adenosine and is metabolized to ribose-1-phosphate and hypoxanthine by purine nucleoside phosphorylase and further to xanthine and uric acid by xanthine deshydrogenase. Consequently, plasma levels of free adenosine in normoxic conditions are extremely low (0.1-0.2 *μ*mol/L) and those of inosine almost undetectable (0.01–0.1 *μ*mol/L).

**Figure 3:**
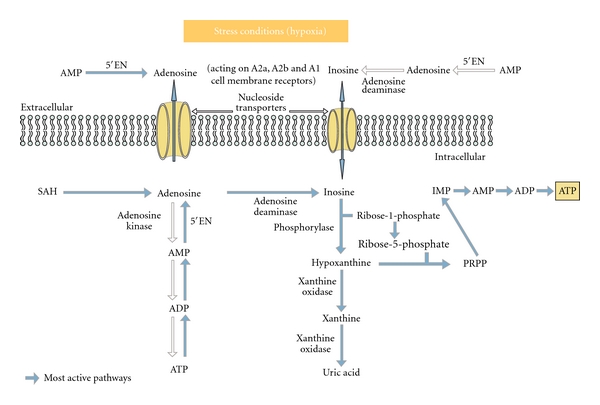
Adenosine and inosine pathways in hypoxic conditions (stress). During *“cellular stress”* conditions (e.g., hypoxia) the production of adenosine increases. Ecto and endo 5′NT enzymes mainly contribute to the production of adenosine in cells. The rate of SAH hydrolysis does not increase much (1.5-fold). When adenosine levels increase, adenosine deamination predominates and inosine reaches high concentration inside the cell. It is then shunted into the extracellular space by bidirectional equilibrative nucleoside transporters and interstitial levels of inosine can rise to greater than 1 mM. An array of evidence now suggests that under hypoxic conditions the phosphorylated ribose moiety of inosine is used for energy repletion through anaerobic glycolysis as an alternative or complementary to glucose. Inosine can generate ATP through the hypoxanthine-IMP-AMP pathway or through the anaerobic pentose pathway (Ribose1P). Hypoxanthine is rephosphorylated to inosine monophosphate using phosphoribosyl pyrophosphate (PRPP), leading to IMP and further AMP. At pH 7.4 ribose-1-phosphates can be converted to ribose-5-phosphate which rebuilds the purine ring system and can restore ATP via the anaerobic pentose pathway.

It is known that under hypoxic condition and contrary to normoxic conditions, myocardial cells produce great quantities of both adenosine and inosine (Figures 2 and 3). Said briefly during hypoxia *adenosine *is utilized mainly for cell protection mechanisms, via stimulation of myocardial cell and endothelial cell adenosine A1, A2, and A3 receptors leading to various biological effects including activation of the mitochondrial K+ ATP dependent channels [30–33] while *inosine* is used for cell energy repletion [8, 11], these two effects being complementary (Figure 3). Well documented is also *adenosine's *ability to inhibit neutrophil cells migration, to reduce free radical production [34, 35], to downregulate pro-inflammatory cytokines (such as TNF-*α*, IL-6, IFN-*γ*, IL-12). Also well known is inosine's ability to attenuate many pro-inflammatory cytokines such as IL-1 and macrophage-inflammatory protein-1 *α* [25, 26]. Importantly, it was observed that the increase in adenosine and inosine formation during myocardial ischemic events is not always sufficient to supply needs. After only a few hours, persistent ATP depletion followed by adenosine and inosine depletion can lead to cell death. This has justified the use, in several human clinical trials (e.g., Attac and Amistad studies, ref. [14–16]), of exogenous adenosine as an adjunct to reperfusion techniques performed within the first 6 hours in patients with acute coronary syndrome. These studies showed interesting outcomes like a significant reduction of infarct size, but unfortunately no clear reduction of patients' mortality and morbidity rates. In addition no significant improvement of cardiac function was demonstrated. Thus, there was some general disappointment at the results, along with the overall feeling that adenosine therapeutic effects and its related methods of use during or after acute myocardial infarction events could be greatly optimized. To achieve this goal different approaches are possible such as the use of specific adenosine agonists or adenosine regulating agents with some of them having shown in ACS animal models interesting preclinical results [36, 37]. Here we tested what can be labeled the “nucleoside approach,” not only because of the various biological activities described above but also because adenosine and inosine are critical energy substrates during acute ischemia (Figures 2 and 3). In this regard it should be noted that adenosine was recently shown to stimulate the AMP-activated protein kinase (AMPK) pathway [38] known to play an important role in restoring the energy balance. AMPK activation increases glucose uptake (leading to pyruvate synthesis and ATP production) and free fatty acids metabolism. Interestingly decrease of free fatty acids uptake and increase of carbohydrates uptake can also be induced by infusion of inosine alone as has been described previously [8]. However, the existence of a link between inosine and the AMPK pathway has not yet been studied.

### 4.2. Optimization of Adenosine Method of Use

Regarding the method of treatment, several studies assessed the effect of adenosine given through the coronary arteries during percutaneous cardiologic interventions. Positive results were obtained when adenosine was given by infusion of 20 minutes or more [39] and negative results were obtained when it was given as a bolus [40]. Consistent with adenosine extremely short half life (a few seconds) it is thus confirmed that bolus administration, as opposed to long infusion times is not effective whatever the intravascular route selected, intra-arterial or intravenous. Moreover, if long infusion times are required to ensure adenosine efficacy it is likely that non invasive routes of administration (intravenous) will appear more adequate in the future compared to intracoronary or intracardiac routes.

Improvement of the method of administration regarding the timing of reperfusion is also critical for cardioprotection. Given the impact of time [41] future studies in the human will have to restrict the administration of drug combination adenosine/inosine employed as a potential protective agent to those patients who can be treated within the first 3–3.5 hours after onset of symptoms.

With regard to the myocardial healing process, the use of subsequent drug combination infusions following the first infusion might also be assessed. Repetition of infusions during the days after myocardial infarction so as to further stimulate angiogenesis could lead to additional beneficial effect. Another justification of repeated administration is the fact that adenosine reduces the release of noradrenaline, the production of endothelin and attenuates activation of the renin-angiotensin system, all of which are thought to cause cardiac hypertrophy and remodeling [42].

### 4.3. Optimization of Adenosine Effects

Regarding the enhancement of adenosine's protecting and repairing activities, we hypothesized that the administration of exogenous inosine, in addition to adenosine, at specific adenosine : inosine dose ratios, might optimize the “nucleoside effect” and improve adenosine's performance. We tested this hypothesis using an animal model that reproduced the same adenosine protocol design as applied in the human for acute coronary syndrome, the same sequence of naturally occurring events, namely, acute ischemia followed by myocardial reperfusion, while the doses of infused nucleosides were correlated to those employed in patients. The presented results showed that the drug combination used at a specific adenosine : inosine 1 : 5 ratio was at least equivalent to adenosine and inosine alone to improve cardiac function (Table 1) and superior to both of them to reduce infarct size (Table 2). In addition whereas the ability of adenosine to stimulate angiogenesis is well documented we also found that inosine had a myocardial proangiogenic activity of its own at least as potent as that of adenosine (Table 2). This angiogenic effect seems to be amplified when the two nuclosides are combined. It has been suggested that inosine could selectively induce angiogenesis in porcine aortic endothelial cells cultivated * in vitro* [43] via fibroblast growth factor (FGF) known as a strong angiogenesis promoter [44]. Thus, although still hypothetical, there is some ground to conduct additional studies for checking if the proangiogenic activity of the adenosine/inosine combination may involve the concurrent stimulation of both VEGF and FGF mediated by adenosine and inosine respectively.

Additionally, it has been suggested that AMPK pathway stimulation by adenosine, besides its important energetic role during acute ischemic conditions, could also actively participate to various steps of the proangiogenic cascade, such as for example endothelial cells migration [38].

In summary, our results indicate that adenosine and inosine are likely to have cell protective and repairing effects that can be enhanced when they are administered simultaneously. It should be highlighted that the two compounds, while chemically similar, use different intracellular pathways, allowing for complementary biological activities without overlapping. This makes their combined use logical and promising for the targeted indication. Further studies are needed to confirm that the drug combination is an optimized therapeutic form of adenosine and thus might be proposed as an adjunct to reperfusion procedures in acute coronary syndrome patients as well as an agent with the ability to accelerate the healing process after myocardial infarction. Final hope is that the addition of all the optimization factors described here may contribute to further reduce myocardial infarction morbidity and mortality rates.

## Figures and Tables

**Table 1:** Echocardiographic LV functional evaluation.

Group	Individuals	LV ejection fraction	SD
Adenosine + Inosine	*n* = 12	0.644	0.087
Adenosine	*n* = 12	0.631	0.086
Inosine	*n* = 12	0.610	0.063
Sham	*n* = 12	0.491	0.123

Ejection fraction at 45 days showed a significant difference between the four groups (Welch's ANOVA *P* = 0.015). C Dunnett test for pairwise comparison was significant for combination versus sham (superiority at 0.05, mean of the difference = 15.2%) and adenosine versus sham (superiority at 0.05, mean of the difference = 14%).

**Table 2:** Infarct size and angiogenesis.

Group	Mean infarct size (mm^3^)	Angiogenesis median index
Adenosine + inosine	1.70 ± 0.18	4
Adenosine	2.60 ± 0.2	2
Inosine	2.62 ± 0.3	3
Sham	3.79 ± 0.45	1.5

The Kruskal Wallis test for the difference between the four groups was significant at the 0.05 level (*P* = 0.02 for the infarct size endpoint and *P* < 0.01 for the angiogenesis index). Using the nonparametric post hoc test (Student Newman Keuls for balanced samples) at the 0.05 level infarct size for both the combination and adenosine groups as compared to the sham group was significantly smaller. The combination drug approach showed a significant and better proangiogenic effect versus adenosine and versus sham groups. At the 0.05 level all treated groups (adenosine, combination, and inosine) were superior to the control group.
